# GFP Scaffold-Based Engineering for the Production of Unbranched Very Long Chain Fatty Acids in *Escherichia coli* With Oleic Acid and Cerulenin Supplementation

**DOI:** 10.3389/fbioe.2019.00408

**Published:** 2019-12-10

**Authors:** Elias Kassab, Norbert Mehlmer, Thomas Brueck

**Affiliations:** Werner Siemens-Chair of Synthetic Biotechnology, Department of Chemistry, Technical University of Munich, Garching, Germany

**Keywords:** VLCFA, *Escherichia coli*, *Arabidopsis thaliana*, self-assembly GFP, fatty acid biosynthesis

## Abstract

Currently, very long chain fatty acids (VLCFAs) for oleochemical, pharmaceutical, cosmetic, or food applications are extracted from plant or marine organism resources, which is associated with a negative environmental impact. Therefore, there is an industrial demand to develop sustainable, microbial resources. Due to its ease of genetic modification and well-characterized metabolism, *Escherichia coli* has established itself as a model organism to study and tailor microbial fatty acid biosynthesis using a concerted genetic engineering approach. In this study, we systematically implemented a plant-derived (*Arabidopsis thaliana*) enzymatic cascade in *Escherichia coli* to enable unbranched VLCFA biosynthesis. The four *Arabidopsis thaliana* membrane-bound VLCFA enzymes were expressed using a synthetic expression cassette. To facilitate enzyme solubilization and interaction of the synthetic VLCFA synthase complex, we applied a self-assembly GFP scaffold. In order to initiate VLCFA biosynthesis, external oleic acid and cerulenin were supplemented to cultures. In this context, we detected the generation of arachidic (20:0), cis-11-eicosenoic (20:1) and cis-13-eicosenoic acid (20:1).

## Introduction

Microbial oils have been recently designated as a sustainable alternative to plant- and animal-based lipids. In that regard, *E. coli* has established itself as a model organism to study and tailor microbial fatty acid biosynthesis using a concerted genetic engineering approach (Janssen and Steinbuchel, 2014). While *E. coli* is not an oleaginous organism *per se* the option for extensive genetic alternation has allowed the generation of respectable product titers (Janssen and Steinbuchel, 2014). However, despite giant strides in increasing the intracellular fatty acid pool and diversifying the profile of natural fatty acids by genetic engineering, currently the ability to generate plant-like fatty acids with industrial demand is very limited (Handke et al., 2011; Janssen and Steinbuchel, 2014; Pfleger et al., 2015). While *E. coli* naturally generates medium chain fatty acids (C6–C12), most genetic engineering efforts have focused either on increasing the natural fatty acid pool or extending the natural profile toward generation of long-chain fatty acids (C13–C19). However, the optimized production of multi-methyl-branched VLCFAs has been successfully reported in *E. coli* by coupling the heterologous pathway for mycocerosic acid production of *M. tuberculosis* with the fatty acid biosynthetic pathway of *E. coli* (Menendez-Bravo et al., 2014, 2016). Currently, there are a few studies that describe the generation of unbranched VLCFAs (C20–C28) that could be applied as food additives or in specialized high value chemical applications, such as performance lubricants (Handke et al., 2011; Pfleger et al., 2015).

More generally, fatty acids (FAs) are carboxylic acids with an aliphatic tail. They are naturally produced by both prokaryotes and eukaryotes and are precursors for the biosynthesis of essential building blocks such as sterols, phospholipids and sphingolipids. Fatty acids are often classified according to their chain length and degree of saturation. In eukaryotic and prokaryotic organisms the chain length can range from short chain fatty acids with aliphatic tails >4 carbons, to VLCFAs with aliphatic tails of >20 carbons. Independent of the chain length, FAs with a single double bond or more are classified as unsaturated FAs, whereas FAs that lack a double bond are classified as saturated FAs (Janssen and Steinbuchel, 2014; Beld et al., 2015, 2016).

VLCFAs are predominantly found in eukaryotic cells. Here they are the precursors for sphingolipid biosynthesis, which is essential for growth (Dickson et al., 2006). In yeast, VLCFAs are reported to be involved in the transport of proteins across membranes by assembling into lipid-protein complexes and play an essential role in the synthesis of the glycosylphosphatidylinositol lipid anchor (Gaigg et al., 2006). In mammals, VLCFAs perform a wide array of physiological functions and are abundant in the myelin sheath of the brain and the lipid barrier of the skin (Jakobsson et al., 2006). In plants, VLCFAs are components neutral lipids, such as triacylglycerol and wax esters, that are used for energy storage in plant seeds and as hydrophobic polymers located on leaf surfaces to prevent water loss and provide resistance to temperature changes (Cassagne et al., 1994; Trenkamp et al., 2004; Dickson et al., 2006; Joubes et al., 2008).

**Figure 1:**
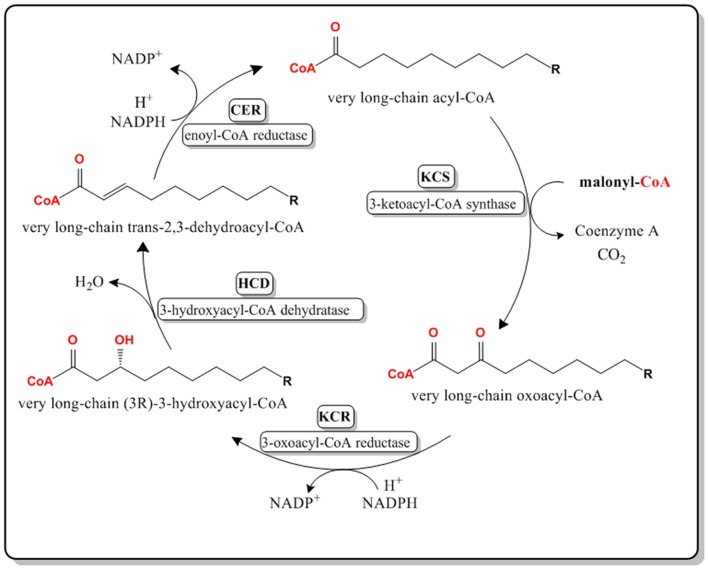
Pathway for the biosynthesis of VLCFAs in the endoplasmic reticulum of *Arabidopsis thaliana* cells. Malonyl-CoA is used as the 2-Carbon elongation unit. *Very long chain* refers to a chain length of 20 carbon atoms or higher.

VLCFAs are synthesized in the endoplasmic reticulum of cells by a membrane-bound enzyme complex that catalyzes the sequential addition of a two-carbon moiety from a malonyl-CoA to a long chain acyl-CoA in the presence of NADH and NADPH (Jakobsson et al., 2006). The complex constitutes four enzymes that perform four distinct reactions that form the elongation cycle (Domergue et al., 2000). 3-ketoacyl-CoA synthases or KCSs catalyze the first step of the cycle through the condensation of a long-chain fatty acyl-CoA with a malonyl-CoA forming a very long chain 3-oxoacyl-CoA (Ghanevati and Jaworski, 2001). The condensing reaction is the rate limiting reaction, therefore the expression level of the condensing enzyme (KCS) highly affects the rate of the overall cycle. The second step of the biosynthetic cycle is the reduction of the very long chain oxoacyl-CoA to (*3R*)*-3*-hydroxyacyl-CoA by a 3-oxoacyl-CoA reductase or KCR. The third step is the dehydration of the (*3R*)*-3*-hydroxyacyl-CoA into a *trans*-2,3-enoyl-CoA by a 3-hydroxacyl-CoA dehydratase or HCD. The final reduction step is catalyzed by a trans-2,3-enoyl-CoA reductase or CER, yielding a two carbon longer acyl-CoA (Zheng et al., 2005; Jakobsson et al., 2006; Joubes et al., 2008; Figure 1).

The chain length and yield of the final product is highly dependent on the KCS expression level and substrate specificity. In *A. thaliana* 20 different genes have been identified encoding a KCS, and are expressed in different tissues and life cycles of the plant depending on the tissue specificity for VLCFAs (Blacklock and Jaworski, 2002; Joubes et al., 2008). Each KCS has a different substrate affinity for saturated and unsaturated long acyl-CoAs of different chain length (Millar and Kunst, 1997). The three other enzymes involved in the elongation process have a broad range of substrate specificity (Fehling and Mukherjee, 1991; Millar and Kunst, 1997).

**Figure 2:**
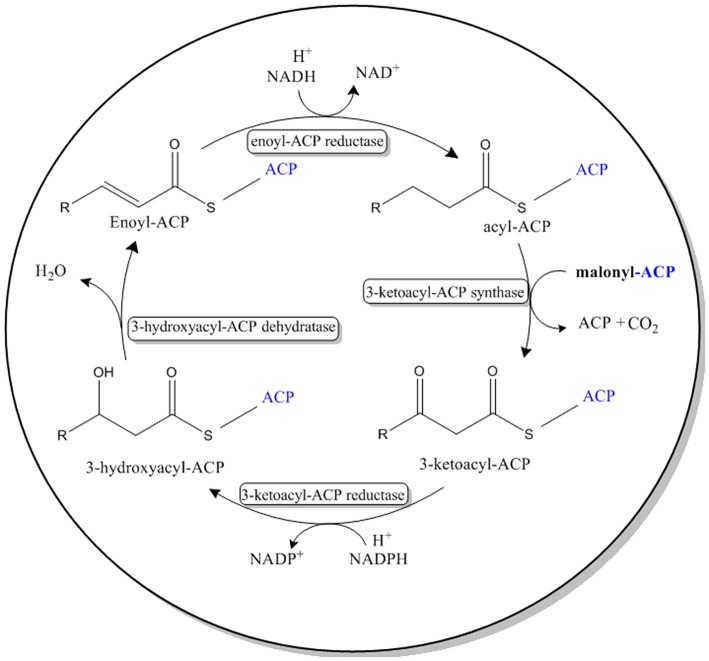
Pathway for the *de novo* biosynthesis of fatty acids in most prokaryotes and eukaryotes. This system can be found in the chloroplast of *A. thaliana* and the cytoplasm of *E. coli*. Malonyl-ACP is used as the 2-Carbon elongation unit.

The four reactions in the VLCFA elongation cycle are similar to that of *de novo* fatty acid biosynthesis, found in prokaryotes and eukaryotes (Jakobsson et al., 2006; Figure 2). However, in *de novo* fatty acid biosynthesis malonyl-ACP is used as a substrate in the condensing reaction.

In *E. coli*, malonyl-CoA synthesized by the acetyl-CoA carboxylase is directly converted to malonyl-ACP by the malonyl-CoA:ACP transacylase (FabD) and directed toward fatty acid biosynthesis (Joshi and Wakil, 1971; Cronan and Waldrop, 2002). The amount of malonyl-CoA synthesized is tightly, transcriptionally and translationally, regulated in *E. coli* (Cronan and Waldrop, 2002). The fatty acid profile ranges from lauric acid (C12:0) up to stearic acid (C18:0), and low amounts of vaccenic acid (C18:1 Δ11). The most abundant fatty acid is palmitic acid (C16:0). Unsaturated fatty acids, mainly composed of palmitoleic and vaccenic acids, constitute 35% of total membrane lipids. Free fatty acids cleaved from the membrane of *E. coli* or taken up from the environment are activated by FadD to Acyl-CoAs and consumed via the beta oxidation pathway (Janssen and Steinbuchel, 2014).

In this study, we have systematically extended our toolbox for engineering of *E. coli* toward the generation of VLCFA's including eicosenoic acid (20:1) and arachidic acid (20:0). We focus on the expression of four enzymes involved in the plant-based VLCFA biosynthesis of *A. thaliana* using a synthetic polycistronic expression cassette in combination with a self-assembly GFP system. The application of the self-assembly GFP system is a highly innovative method to enable simultaneous solubilization and guided interaction of the plant-derived fatty acid biosynthesis enzyme systems (Cabantous et al., 2005; Cabantous and Waldo, 2006; Venning-Slater et al., 2014; Xie et al., 2017). Furthermore, oleic acid and cerulenin were required to initiate VLCFA biosynthesis.

## Materials and Methods

### Genes and Plasmids

Nucleotide sequences of *KCS18* (Gene ID: 829603), *KCR1* (Gene ID: 843098), *PAS2* (Gene ID: 830912), and *CER10* (Gene ID: 824702) were obtained from The Arabidopsis Information Resource (TAIR), on www.arabidopsis.org database. Nucleotide sequences for the self-assembly GFP were obtained from the published sequences of Cabantous et al. (2005). The putative transmembrane domain of only *KCR1* (NP_564905.1) was predicted using the TMHMM Server v. 2.0 (Krogh et al., 2001) and removed from the mature DNA sequence. The mature sequences were codon-optimized for expression in *E. coli* and chemically synthesized by Eurofins Scientific. The RBS calculator software tool was used to design and evaluate the relevant ribosomal binding sites (RBS) (Salis et al., 2009; Espah Borujeni et al., 2014). The cloned genes were confirmed by sequencing (Eurofins Scientific). All primers were synthesized by Eurofins Scientific and all plasmids were obtained from Novagen/Merk Millipore.

### Bacterial Strains and Growth Conditions

All bacterial strains used were obtained from Merk Millipore. For cloning and plasmid amplification, *E. coli* DH5 alpha strain was used. *E. coli* BL21 (DE3) strain was used for expression and fatty acid production. Minimal M9 media [1 g L^−1^ NH_4_Cl, 0.5 g L^−1^ NaCl, 3 g L^−1^ KH_2_PO_4_, 6 g L^−1^ Na_2_HPO_4_, 0.493 g L^−1^ MgSO_4_·7H_2_O, 0.011 g L^−1^ CaCl_2_, 0.42 g L^−1^ FeCl_3_- 6·H_2_O] supplemented with 0.4% glucose and a pH of 6.9 was used for the shake flask studies, clones were grown at 37°C with the appropriate antibiotics (Kanamycin 50 μg/mL and Chloramphenicol 34 μg/mL) and induced at an OD_600_ of 0.6 with 0.05 mM IPTG (isopropyl-β-D-thiogalactopyranoside).

### Fluorescence Microscopy

For microscopy, cells were washed and resuspended in ddH_2_O. Microscope photographs were acquired on an Axio Lab. A1, fluorescence microscope equipped with an Axio Cam ICm1 (Zeiss, Oberkochen, Germany).

### Fatty Acid Methylation

Samples taken from shake flask and fermentation studies were centrifuged and subsequently washed twice with ddH_2_O. Subsequently, the pellets were then lyophilized and equal amounts of dry cell weight were weighed and taken for analysis. Methanol transesterification according to the protocol of Griffiths et al. (2010) was used to directly convert dry cell biomass to fatty acid methyl esters (FAMES).

### Fatty Acid Analysis

One microliter of each sample was injected into Gas Chromatograph-Flame Ionization Detector (GC-FID) for separation and quantification of the FAMEs. GC–MS was performed with the Thermo Scientific™ TRACE™ Ultra Gas Chromatograph instrument coupled to a Thermo DSQ™ II mass spectrometer and the Triplus™ Autosampler injector. MS was performed in positive ion mode. The analysis was carried out using a Stabilwax^®^ fused silica capillary column (30 m × 0.25 mm, with a film thickness of 0.25 μm). The run was under an optimized temperature as follows: initial column temperature 50°C, programmed to increase at a rate of 4 °C/min up to a final temperature of 250°C. Hydrogen was used as the carrier gas at a flow rate of 35 mL/min with constant flow compensation. Additionally, Shimadzu GC-2010 Plus gas chromatograph with flame ionization detector (FID) was also used for fatty acid analysis. One microliter sample was injected via an AOC-20i auto injector (Shimadzu) on to a Phenomenex ZB-WAX column (length 30 m, 0.32 mm ID, 0.25 μm df). The column was heated up with 5°C min-1 to 240°C maintained for 5 min. Hydrogen was used as carrier gas with a flow rate of 3 mL min-1 and constant flow compensation. FAMEs Marine Oil Standard (Marine Oil FAME Mix, RESTEK USA) was used as a standard reference, containing 20 components from C14:0 until C24:1. Glyceryl trinonadecanoate (C19:0 TAG) (Sigma, Germany) was used as internal standard to determine esterification efficiency. Individual FAME concentrations were based on peak areas relative to methyl non-adecanoate (C19:0) (Griffiths et al., 2010).

### Fermentation

The DASGIP^®^ 1.3 L parallel reactor system (Eppendorf AG) was used to perform parallel fermentations. A modified M9 media [8 g L^−1^ NH_4_Cl, 13.3 g L^−1^ KH_2_PO_4_, 1.2 g L^−1^ MgSO_4_·7H_2_O, 0.42 g L^−1^ FeCl_3_-6·H_2_O, 20 g L^−1^ glucose] was used as batch media and supplemented with 1 mL 100 × trace elements solution (5 g L^−1^ EDTA; 0.83 g L^−1^ FeCl_3_- 6·H_2_O; 84 mg L^−1^ ZnCl_2_, 13 mg L^−1^ CuCl_2_-2·H_2_O, 10 mg L^−1^ CoCl_2_-2·H_2_O, 10 mg L^−1^ H_3_BO_3_, and 1.6 mg L^−1^ MnCl_2_-4·H_2_O) and the proper antibiotics. Fermenters were inoculated with an overnight pre-culture with a starting OD_600_ of 0.1. The cultivation temperature was kept constant at 30 °C. Initial stirring velocity and airflow was set to 200 rpm and to 0.2 volumes of air per volume of medium per min (vvm), respectively. Dissolved oxygen was kept at 30% of the maximum dissolved oxygen concentration (mg/L) by successive increases of the stirrer velocity, the oxygen proportion, and eventually the airflow. A pH value of 7.00 was controlled by the addition of 6 M aqueous NaOH. A pH value shift above 7.05 initiated a feed shot of 40 mL. The feed solution consisted of 500 g L^−1^ glucose, 5 g L^−1^ oleic acid, 20 g L^−1^ MgSO_4_·7H_2_O, 2 mg L^−1^ thiamine–HCl, 16 mL 100 × trace elements solution (pH = 7.00). Samples were taken at different time points to determine the OD_600_. Once the clones reached the stationary phase of growth, they were induced with 0.05 mM IPTG. Five micromolar Cerulenin (Cayman chemicals, USA) was added 24 h after induction with IPTG.

## Results and Discussion

### KCSs in *E. coli* System

In order to enable the recombinant production of VLCFA in *E. coli* BL21 (DE3), we constructed and expressed the characterized *Arabidopsis thaliana* VLCFA multi-enzyme elongase system using a synthetic expression cassette. The first step of fatty acid elongation is the condensation of a fatty-acyl-CoA with a malonyl-CoA catalyzed by 3-ketoacyl-CoA synthase (Ghanevati and Jaworski, 2001). Twenty-one gene-homologs of the 3-ketoacyl-CoA synthase have been identified in *A. thaliana* (Costaglioli et al., 2005). The different KCS homologs were classified into 8 subclasses according to their phylogeny, duplication history, genomic organization, protein topology and 3D modeling (Joubes et al., 2008). Of the 21 KCS homologs, only eight (*KCS1, KCS5, KCS9, KCS11, KCS14, KCS17, KCS18*, and *KCS20*) have been tested and demonstrated to encode proteins able to catalyze VLCFA production (Trenkamp et al., 2004; Blacklock and Jaworski, 2006; Paul et al., 2006). Furthermore, *KCS1, KCS6* and *KCS10* were reported to be the most expressed of all the homologs, suggesting that the other homologs are functionally redundant (Joubes et al., 2008). Three genes, *KCS1, KCS6*, and *KCS18* each falling within a different subclass, were individually expressed in *E. coli* BL21 (DE3). Out of the 21 homologs, *KCS1, KCS6, and KCS18* were chosen based on the differences in substrate specificity, biological function, and expression patterns in *A. thaliana* (Blacklock and Jaworski, 2006; Joubes et al., 2008; Table 1). Respectively, we have performed a fatty acid analysis in order to determine any changes in the fatty acid profile of *E. coli*. Three pET28a vectors with T7 promoters, each harboring one of the KCS homologs, were used for expression in BL21 (DE3). Clones were cultivated at 37°C and 30°C in both LB and M9 minimal media respectively. After induction with 0.05 mM IPTG, we performed a lipid analysis where we found that the individual expression of three 3-ketoacyl-CoA synthases (*KCS1, KCS6*, and *KCS18*) in *E. coli* BL21 (DE3) did not result in detectable amounts of VLCFA (Supplementary Material). Since wild type *E. coli* is endogenously not capable of generating VLCFAs, the entire *A. thaliana* elongase multi-enzyme complex was cloned to enable VLCFA biosynthesis independent of the *E. coli* native fatty acid synthase.

**Table 1:** Properties of A. *thaliana* ketoacyl-CoA synthases used in this study.

**KCS**	**Biological function**	**Expression patterns**	**Substrate specificity**	**Product**	**References**
KCS1	Required for cuticular wax production	Expressed in all tissues; Highest expression in siliques, flowers and stems	C16:0, C16:1, C18:0, C18:1 (very low activity) and C20:1	C20:0, C20:1, C22:0, C22:1, C24:0 and C26:0	Todd et al., 1999; Joubes et al., 2008; Haslam and Kunst, 2013
KCS6	Required for cuticular wax production	Expressed in all tissues; specialized expression in epidermis	C22:0, C24:0 and C26:0	C24:0, C26:0 and C28:0	Hooker et al., 2002; Haslam and Kunst, 2013; Janssen and Steinbuchel, 2014
KCS18 (FAE1)	Required for the production of VLCFA for TAG storage in seeds	Highest in seeds; Found in carpels and siliques	C16:0, C16:1, C18:0, C18:1; lower activity with 20:0 and 20:1	C18:1; C20:0; C20:1; C22:0; C22:1; Low amounts of C24 and C26	James et al., 1995; Joubes et al., 2008; Haslam and Kunst, 2013

### Synthetic Design of an Elongase Expression Cassette Using RBS Prediction

Since we could not detect a change in fatty acid distribution by the individual expression of the three KCSs homologs, we referred back to literature. Based on the collected information *KCS18* was chosen as the first enzyme in the complex due to its broader range of substrate specificity compared to equivalent homologs (Ghanevati and Jaworski, 2001; Blacklock and Jaworski, 2002; Paul et al., 2006; Joubes et al., 2008). The additional three enzymes (KCR1, PAS2, and CER10) involved in the elongase complex have a broad range of specificity and are expressed in all tissues exhibiting VLCFA (Fehling and Mukherjee, 1991; Millar and Kunst, 1997). Two of these enzymes (KCR1, CER10) involved in the complex are well characterized in A. *thaliana*: KCR1, 3-ketoacyl-CoA reductase, performs the second step of the elongation process (Xu et al., 1997, 2002; Beaudoin et al., 2002), PAS2, 3-hydroxacyl-CoA dehydratase, the least characterized of the four, catalyzes the third step (Bach et al., 2008; Roudier et al., 2010) and CER10, trans-2,3-enoyl-CoA reductase, performs the last step of the elongation process. PAS2 is known to interact with CER10 in the ER of *A. thaliana* (Zheng et al., 2005). The ribosomal binding site of each gene on the VLCFA expression cassette was predicted using the RBS Calculator software (Supplementary Material) to control translation initiation and protein expression rates (Salis et al., 2009; Espah Borujeni et al., 2014). Since KCS18 catalyzes the rate limiting step in VLCFA biosynthesis, the synthetic RBS sequence of *KCS18* was calculated to yield a double translation initiation rate and double protein expression level to that of *KCR1, PAS2*, and *CER10*. The synthetic RBS sequences of *KCR1, PAS2*, and *CER10* were calculated to yield equimolar expression levels of each protein. The expression cassette was further evaluated for protein expression using operon expression calculator (Salis et al., 2009; Espah Borujeni et al., 2014).

### Design of GFP-Based Scaffold Elongase Expression Cassette

**Figure 3:**
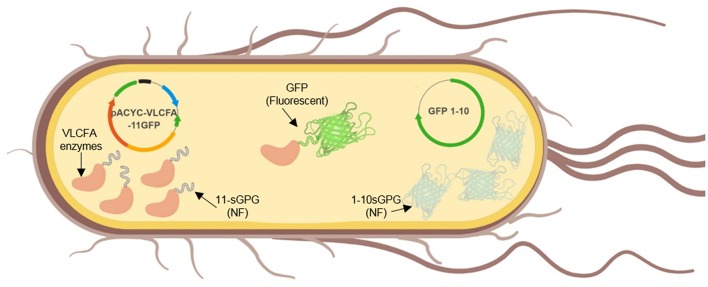
Graphical illustration of the *in vivo* expression of self-assembly GFP and the VLCFA biosynthesis enzymes.

Green Fluorescent Protein (GFP) is a barrel-shaped protein consisting of 11 Betta-sheets. Previous studies have developed a mutated version of GFP, termed split-GFP or self-assembly GFP, where the gene encoding the first 10 beta sheets (GFP1-10) and the gene encoding the last beta sheet (GFP11) are expressed separately but are able to self-assemble and fluoresce (Cabantous et al., 2005; Cabantous and Waldo, 2006). Self-assembly GFP has been previously reported to enhance protein solubility, reduce formation of bacterial inclusion bodies and for the immobilization of various enzymes in recombinant *E. coli* BL21 (DE3) (Cabantous and Waldo, 2006; Venning-Slater et al., 2014). It has also been applied for topology analyses of membrane-bound enzymes in N. *benthamiana*, without affecting their biological functionality (Xie et al., 2017). In order to enhance solubilization and immobilize the VLCFA elongase system in *E. coli*, each gene in the VLCFA expression cassette was genetically tagged with the *GFP11* sequence at its N-terminus. Concomitantly, the *GFP1-10 was* cloned and co-expressed on a separate *pET28a* vector (Figure 3). Notably, due to the several repetitive regions implemented on each gene, cloning was very challenging. Therefore, the expression cassette was split into five fragments and chemically synthesized. The first two fragments were joined via overlap PCR and cloned into an empty pACYC vector. The remaining fragments were added sequentially to the pACYC vector containing the first two fragments using a series of restriction and ligations with *SapI* and *SpeI* restriction enzymes respectively.

### Expression in BL21 (DE3)

**Figure 4:**
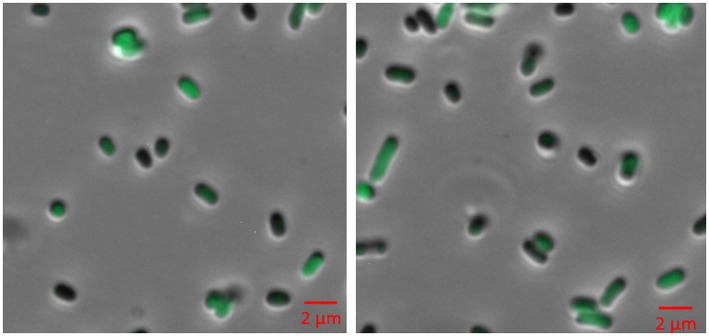
Fluorescence microscopy of BL21 (DE3) cells expressing pACYC-VLCFA-11GFP and pET28a-1-10GFP. Cells were cultured in LB media and induced with 0.05 mM IPTG for 6 h. For microscopy, cells were washed and re-suspended in ddH_2_O.

The synthetic *VLCFA* expression cassette and in combination with the self-assembly GFP constructs were co-expressed in *E. coli* BL21 (DE3). The cultivation was conducted with minimal M9 media supplemented with 0.5% glucose at 30°C. Cultures were induced with 0.05 mM IPTG at an OD_600_ of 0.6 (Figure 4). Samples were collected 24 h after induction, the lipid fraction was extracted and methylated. After analysis, we could not detect VLCFA production (Supplementary Material).

**Figure 5:**
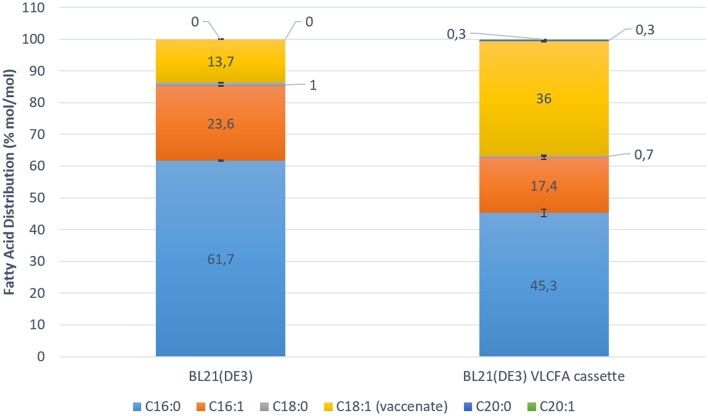
Fatty acid distribution mol/mol of control *Escherichia coli* BL21 (DE3) expressing pACYC-11-saGFP and pET28a-1-10saGFP vs. BL21 (DE3) expressing pACYC-VLCFA-11GFP and pET28a-1-10GFP in 100 ml shake flask studies. The overall increase in C18:1 fatty acids is highly noticeable. The BL21 (DE3) VLCFA cassette profile clearly shows the production of longer than C18 fatty acids (C20:0 and C20:1) which are absent in the control.

KCS18 is reported to have high affinity for oleyl-CoA (James et al., 1995; Paul et al., 2006; Sun et al., 2013). Additionally, *E. coli* is reported to be able to grow on oleic acid as sole carbon source (Janssen and Steinbuchel, 2014). Hence, external oleic acid is transported from the media into the cell via the membrane transport protein (FadL), which is then activated into oleoyl-CoA by FadD (Campbell and Cronan, 2002; Lepore et al., 2011). In order to increase the amount acyl-CoAs, M9 media was supplemented with 0.5 % oleic acid. Cultures were induced with 0.05 mM IPTG when OD_600_ of 0.6 was reached. In order to increase malonyl-CoA concentrations as the second substrate required for fatty acid elongation, 5 μM cerulenin was added to the cultures at the stationary phase (van Summeren-Wesenhagen and Marienhagen, 2015). Cerulenin binds irreversibly to the native *E. coli* beta-ketoacyl-ACP synthases (FabB and FabF) leading to the accumulation of malonyl-CoA (Janssen and Steinbuchel, 2014). Subsequently, cerulenin also irreversibly binds to KCS18, however the concentration of cerulenin used should not completely inhibit the function of KCS18 (Schneider et al., 1993). After 24 h of induction with IPTG, samples were collected and analyzed for their lipid content by GC-FID. In samples expressing the VLCFA expression cassette, we detected minor peaks corresponding to arachidic acid (20:0) methyl ester and eicosanoic acid (20:1) methyl ester. Quantitative analysis revealed a change in the fatty acid distribution of the clone expressing the VLCFA cassette (Figure 5).

### Fermentation and Scale Up

**Figure 6:**
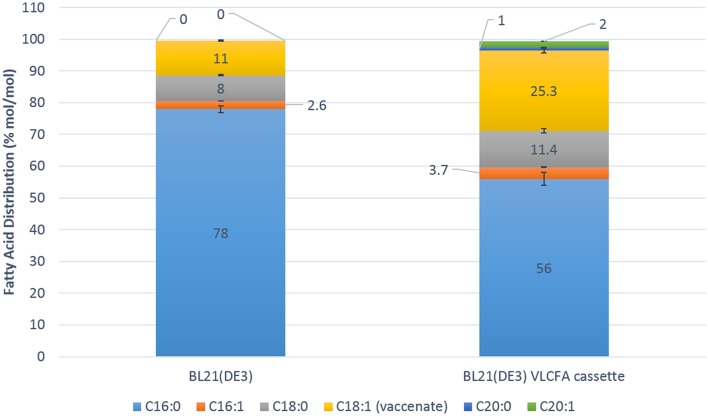
Fatty acid distribution mol/mol of the 1.3 L Fermentation of the control *Escherichia coli* BL21 (DE3) expressing pACYC-11-saGFP and pET28a-1-10saGFP vs. BL21 (DE3) expressing pACYC-VLCFA-11GFP and pET28a-1-10GFP. The BL21 (DE3) VLCFA cassette profile clearly shows the production of longer than C18 fatty acids (C20:0 and C20:1) which are absent in the control in addition to increases in stearic and vaccenic acid.

Fed batch fermentation experiments were carried out in a 1.3 L parallel fermenter in order to investigate the full potential of the recombinant VLCFA system in an optimized microbial system. The feed was supplemented with 0.5 % oleic acid and 0.05 mM IPTG was used for induction. Five micromolar cerulenin was added to the cultures 24 h after induction with IPTG. Quantitative analyses using GC-FID revealed a change in the fatty acid distribution of the clone harboring the VLCFA cassette. Interestingly, stearic acid (18:0) contributed 11.4% mol/mol of the total fatty acid pool, while vaccenic acid (18:1) made up 25.3% mol/mol. The total C18 fatty acid fraction was therefore 36.7% mol/mol of the total fatty acid pool. Arachidic acid (20:0) comprised 1% mol/mol of the total fatty acid pool and eicosenoic acid (20:1) comprised 2% mol/mol of the total fatty acid pool (Figure 6). We estimate that we obtained 6 mg of total VLCFAs per liter of culture with an estimated productivity of 0.25 mg/l/h.

**Figure 7:**
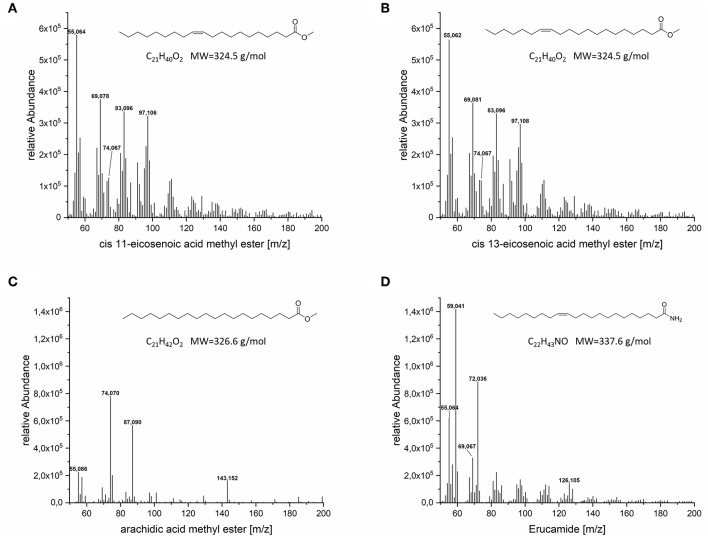
MS spectra of the VLCFA peeks detected in GC-FID. **(A)** cis-11-eicosenoic acid methyl ester, **(B)** cis-13-eicosenoic acid methyl ester, **(C)** eicosanoic acid methyl ester, **(D)** erucamide.

GC-MS could confirm the presence of arachidic acid methyl ester, eicosenoic acid methyl ester and surprisingly erucamide, an amide of erucic acid (C22:1), in the lipid fraction of the VLCFA clone (Figure 7). Two separate signals referring to cis-11-eicosenoic acid methyl ester and cis-13-eicosenoic acid methyl ester were also identified (Supplementary Material). Cis-11-eicosenoic acid (20:1) is the two carbon elongated version of the cis-9-octadecenoic acid (oleic acid 18:1), whereas cis-13-eicosenoic acid (20:1) is the derivative of the cis-11-octadecenoic acid (vaccenic acid 18:1), which is natively produced *de novo* in *E*. coli. We also observed and confirmed the presence of erucamide in the lipid fraction of our clones. Erucic acid (C22:1) is the 4 carbon elongated derivative of oleic acid. While we currently cannot explain the presence of the amidated version of erucic acid, we are conducting a detailed metabolomic study to elucidate its potential biosynthetic origin.

Further optimization of upstream and downstream processes is required to efficiently produce and increase VLCFA titers in *E. coli*. As *E. coli* is not a lipid-accumulating organism *per se*, the biosynthetically generated fatty acids are commonly transported and stored in the membrane as diacylglycerols (DAGs). It is currently uncertain whether *E. coli* can incorporate VLCFAs in its membrane or activate VLCFAs to be degraded via the beta oxidation pathway.

## Conclusion

VLCFAs are essential building blocks in most eukaryotic and multicellular organisms. VLCFAs and their derivatives are crucial for plant survival and are important for the development and maintenance of nervous and cardio-vascular system in mammals. The high value applications of VLCFA and their derivatives has been reported across the chemical, pharmaceutical, food and cosmetic industries. The ease and the extensive genetic manipulation of *E. coli* for the production of high value chemicals have been shown to be a sustainable alternative to plant-based platforms. In this study, we cloned and expressed the *A. thaliana* VLCFA synthase complex in *E. coli* for the generation of arachidic and eicosenoic acid by supplementing oleic acid and cerulenin. The solubilization of the membrane associated, plant-derived enzyme cascade that enabled VLCFA production in *E. coli* was facilitated by application of an innovative self-assembly GFP system. In that context, the fusion of individual enzyme activities that are part of the heterologous VLCFA biosynthesis complex, to the engineered GFP system also facilitated the interaction of relevant enzyme systems within the *E. coli* cell.

Since wild type *E. coli* is endogenously not capable of generating VLCFAs, their metabolism, which may include further enzymatically mediated functionalization, is hitherto not established. Further systems biology analysis is required in order to assess the impact of VLCFAs production on *E. coli* metabolism. In this context, the detection of erucamide in this study, suggested that there is a cross talk between, the endogenous, primary amino acid biosynthesis and the heterologous VLCFA biosynthetic cascade. While the biosynthesis of primary amides is not well-characterized, their natural occurrence in fungi, plants and mammals has been well-documented (Sun et al., 2016; Li et al., 2017; Kim et al., 2018). While, a discrete investigation of metabolic effects is beyond the scope of this study, we are currently addressing the observed metabolic effects by a concerted systems biology approach. Furthermore, additional genetic strategies are required to further increase malonyl-coA levels and to create a metabolic sink for recombinantly generated fatty acids, thereby increasing space-time and total concentrations of tailored fatty acid products.

## Data Availability Statement

All datasets generated for this study are included in the article/Supplementary Material. Additional data required is available from the corresponding author on reasonable request.

## Author Contributions

EK and TB conceived the project. EK designed and performed the experiments. EK and NM analyzed the data. EK, NM, and TB prepared the manuscript. TB and NM supervised the whole work.

### Conflict of Interest

The authors declare that the research was conducted in the absence of any commercial or financial relationships that could be construed as a potential conflict of interest.
