# Supplementary material for: GFP Scaffold-Based Engineering for the Production of Unbranched Very Long Chain Fatty Acids in Escherichia coli With Oleic Acid and Cerulenin Supplementation
Source: Front Bioeng Biotechnol. 2019 Dec 10;7:408. doi: 10.3389/fbioe.2019.00408 (PMC6914682; doi:10.3389/fbioe.2019.00408)
Supplement: Supplementary file 1 [file Data_Sheet_1.PDF]

Supplementary Material

Table S1. List of Ribosomal Binding Sites sequences used in the synthetic expression cassette in this study. RBS1: RBS for *KCS18*; RBS2: RBS for *KCR1*; RBS3: RBS for *PAS2*; RBS4: RBS for *CER10*.

Name	Sequence	Reference
RBS1	TGTAATATAATATTCTTCTAAGGGGGTCGTAG	(Salis et al., 2009; Espah Borujeni et al., 2014)
RBS2	GAGGACAGTGAGCAGAGAGGAGAGA	(Salis et al., 2009; Espah Borujeni et al., 2014)
RBS3	TAAAATTGACAAATAAGATGGGAGG	(Salis et al., 2009;Espah Borujeni et al., 2014)
RBS4	TAAATTTATAGGTTTTATTTAGGAGGGTAGAGAT	(Salis et al., 2009;Espah Borujeni et al., 2014)

Table S2. List of genes and their amino acid sequences used in this study. Only the putative transmembrane domain of *KCR1 (highlighted in red)* was predicted and cleaved off.

Synthesized DNA	Amino acid Sequence	Reference
KCS18	MTSVNVKLLYRYVLTNFFNLCLFPLTAFLAGKASRLTINDLHNFL SYLQHNLITVTLLFAFTVFGLVLYIVTRPNPVYLVDYSCYLPPPHL KVSVSKVMDIFYQIRKADTSSRNVACDDPSSLDFLRKIQERSGLG DETYSPEGLIHVPPRKTFAASREETEKVIIGALENLFENTKVNPREI GILVVNSSMFNPTPSLSAMVVNTFKLRSNIKSFNLGGMGCSAGVI AIDLAKDLLHVHKNTYALVVSTENITQGIYAGENRSMMVSNCLF RVGGAAILLSNKSGDRRRSKYKLVHTVRTHTGADDKSFRCVQQE DDESGKIGVCLSKDITNVAGTTLTKNIATLGPLILPLSEKFLFFATF VAKKLLKDKIKHYYVPDFKLAVDHFCIHAGGRAVIDELEKNLGL SPIDVEASRSTLHRFGNTSSSSIWYELAYIEAKGRMKKGNKAWQI ALGSGFKCNSAVWVALRNVKASANSPWQHCIDRYPVKIDSDLSK SKTHVQNGRS	This work
KCR1	MEICTYFKSQPTWLLILFVLGSISIFKFIFTLLRSFYIYFLRPSKNLRR YGSWAIITGPTDGIGKAFAFQLAQKGLNLILVARNPDKLKDVSDSI RSKYSQTQILTVVMDFSGDIDEGVKRIKESIEGLDVGILINNAGMS YPYAKYFHEVDEELINNLIKINVEGTTKVTQAVLPNMLKRKKGAI INMGSGAAALIPSYPFYSVYAGAKTYVDQFTKCLHVEYKKSGIDV QCQVPLYVATKMTKIRRASFLVASPEGYAKAALRFVGYEAQCTP YWPHALMGAVVSALPESVFESFNIKRCLQIRKKGLQKDSMKKE	NP_564905.1
Mature *KCR1*	MTLLRSFYIYFLRPSKNLRRYGSWAIITGPTDGIGKAFAFQLAQKG LNLILVARNPDKLKDVSDSIRSKYSQTQILTVVMDFSGDIDEGVKR IKESIEGLDVGILINNAGMSYPYAKYFHEVDEELINNLIKINVEGTT KVTQAVLPNMLKRKKGAIINMGSGAAALIPSYPFYSVYAGAKTY VDQFTKCLHVEYKKSGIDVQCQVPLYVATKMTKIRRASFLVASPE GYAKAALRFVGYEAQCTPYWPHALMGAVVSALPESVFESFNIKR CLQIRKKGLQKDSMKKE	This work
PAS2	MAGFLSVVRRVYLTLYNWIVFAGWAQVLYLAITTLKETGYENV YDAIEKPLQLAQTAAVLEILHGLVGLVRSPVSATLPQIGSRLFLTW GILYSFPEVRSHFLVTSLVISWSITEFPITTWIVEIIRYSFFGFKEALG FAPSWHLWLRYSSFLLLYPTGITSEVGLIYLALPHIKTSEMYSVRM PNILNFSFDFFYATILVLAIYVPGSPHMYRYMLGQRKRALSKSKRE	This work
CER10	MKVTVVSRSGREVLKAPLDLPDSATVADLQEAFHKRAKKFYPSR QRLTLPVTPGSKDKPVVLNSKKSLKEYCDGNNNSLTVVFKDLGA QVSYRTLFFFEYLGPLLIYPVFYYFPVYKFLGYGEDCVIHPVQTYA MYYWCFHYFKRILETFFVHRFSHATSPIGNVFRNCAYYWSFGAYI	This work

	AYYVNHPLYTPVSDLQMKIGFGFGLVCQVANFYCHILLKNLRDPS GAGGYQIPRGFLFNIVTCANYTTEIYQWLGFNIATQTIAGYVFLAV AALIMTNWALGKHSRLRKIFDGKDGKPKYPRRWVILPPFL	
1-10 sGFP	MGGTSSKGEELFTGVVPILVELDGDVNGHKFSVRGEGEGDATIGK LTLKFICTTGKLPVPWPTLVTTLTYGVQCFSRYPDHMKRHDFFKS AMPEGYVQERTISFKDDGKYKTRAVVKFEGDTLVNRIELKGTDF KEDGNILGHKLEYNFNSHNVYITADKQKNGIKANFTVRHNVEDG SVQLADHYQQNTPIGDGPVLLPDNHYLSTQTVLSKDPNEKGT	(Cabantous et al., 2005;Cabantous and Waldo, 2006)
11 sGFP	MTSGSDGGSGGGSTSRDHMVLHEYVNAAGIT	(Cabantous et al., 2005;Cabantous and Waldo, 2006)

Table S3. Fatty acid distribution in µg/g Dry cell weight and mol/mol of the control *Escherichia coli* BL21 (DE3) expressing pET28a- versus BL21 (DE3) expressing pET28a-KCS1, pET28a-KCS6 and pET28a-KCS18 of the 100 ml shake flask studies. Samples were collected 24 hours after induction with 0.05 mM IPTG. All fatty acid values are the average of at least three biological replicates with the associated standard deviation indicated.

	**Fatty acid distribution µg/g DCW**							
**Fatty Acid**	BL21(DE3) pET28a-		BL21(DE3) pET28a-KCS1		BL21(DE3) pET28a-KCS6		BL21(DE3) pET28a-KCS18	
**C16:0**	381.7	±18.8	313.7	±9.6	431.3	±33.9	352.6	±9.11
**C16:1**	113.4	±5.1	93.6	±1.6	124.7	±2.0	111.9	±2.8
**C18:0**	15.1	±0.8	12.8	±0.81	12.1	±1.7	12.4	±0.3
**C18:1 (vaccenic)**	108.1	±5.2	94.9	±4.5	102.7	±3.9	98.7	±4.8
**C20:0**	-	-	-	-	-	-	-	-
**C20:1**	-	-	-	-	-	-	-	-
**C20:2**	-	-	-	-	-	-	-	-
**C20:3**	-	-	-	-	-	-	-	-
**C20:4**	-	-	-	-	-	-	-	-
**C20:5**	-	-	-	-	-	-	-	-
**C22:0**	-	-	-	-	-	-	-	-
**C22:1**	-	-	-	-	-	-	-	-
**C22:6**	-	-	-	-	-	-	-	-
**C24:0**	-	-	-	-	-	-	-	-
**C24:1**	-	-	-	-	-	-	-	-
**Total**	618.29	±30.0	514.95	±16.6	670.91	±41.6	575.54	±17.1

Table S3. Continued

	Fatty acid distribution %			
**Fatty Acid**	BL21(DE3) pET28a-	BL21(DE3) pET28a-KCS1	BL21(DE3) pET28a-KCS6	BL21(DE3) pET28a-KCS18
**C16:0**	61.7	64.3	60.9	61.3
**C16:1**	18.3	18.6	18.2	19.4
**C18:0**	2.4	1.8	2.5	2.2
**C18:1 (vaccenic)**	17.5	15.3	18.4	17.1
**C20:0**	-	-	-	-
**C20:1**	-	-	-	-
**C20:2**	-	-	-	-
**C20:4**	-	-	-	-
**C20:3**	-	-	-	-
**C20:5**	-	-	-	-
**C22:0**	-	-	-	-
**C22:1**	-	-	-	-
**C24:0**	-	-	-	-
**C22:6**	-	-	-	-
**C24:1**	-	-	-	-
**Total**	100	100	100	100

Table S4. Fatty acid distribution in µg/20 mg Dry cell weight and mol/mol of the control *Escherichia coli* BL21 (DE3) expressing pACYC-11-saGFP and pET28a-1-10saGFP versus BL21 (DE3) expressing pACYC-VLCFA-11GFP and pET28a-1-10GFP of the 100 ml shake flask studies. Samples were collected 24 hours after induction with IPTG. All fatty acid values are the average of at least three biological replicates with the associated standard deviation indicated.

	**Fatty acid distribution µg/g DCW**				**Fatty acid distribution %**	
**Fatty Acid**	BL21(DE3) control		BL VLCFA cassette		BL21(DE3) control	BL VLCFA cassette
**C16:0**	161.02	± 1.79	178.47	± 12.36	61.7	45.3
**C16:1**	61.68	± 0.95	68.53	± 4.86	23.6	17.4
**C18:0**	2.65	± 0.03	2.91	± 0.18	1.0	0.7
**C18:1 (vaccenic)**	35.50	± 0.50	142.08	± 2.84	13.6	36.0
**C20:0**	-	-	1.32	± 0.03	-	0.3
**C20:1**	-	-	1.01	± 0.02	-	0.3
**C20:2**	-	-	-	-	-	-
**C20:3**	-	-	-	-	-	-
**C20:4**	-	-	-	-	-	-
**C20:5**	-	-	-	-	-	-
**C22:0**	-	-	-	-	-	-
**C22:1**	-	-	-	-	-	-
**C22:6**	-	-	-	-	-	-
**C24:0**	-	-	-	-	-	-
**C24:1**	-	-	-	-	-	-
**Total**	260.86	± 3.28	394.32	± 20.28	100	100

Table S5. Fatty acid distribution in µg/20 mg Dry cell weight and mol/mol of the control *Escherichia coli* BL21 (DE3) expressing pACYC-11-saGFP and pET28a-1-10saGFP versus BL21 (DE3) expressing pACYC-VLCFA-11GFP and pET28a-1-10GFP of the 1.3L Fermentation studies. Samples were collected 24 hours after induction with IPTG. All fatty acid values are the average of at least three biological replicates with the associated standard deviation indicated.

	**Fatty acid distribution µg/20 mg DCW**				**Fatty acid distribution %**	
**Fatty Acid**	BL21(DE3) control		BL VLCFA cassette		BL21(DE3) control	BL VLCFA cassette
**C16:0**	288.02	± 10.67	349.67	± 20.35	78.0	56.5
**C16:1**	9.60	± 0.36	22.61	± 0.95	2.6	3.7
**C18:0**	30.03	± 1.35	70.33	± 3.40	8.1	11.4
**C18:1 (vaccenic)**	41.65	± 2.21	156.40	± 6.78	11.3	25.3
**C20:0**	-	-	4.28	± 0.1	-	1.0
**C20:1**	-	-	15.75	± 0.2	-	2.0
**C20:2**	-	-	-	-	-	-
**C20:3**	-	-	-	-	-	-
**C20:4**	-	-	-	-	-	-
**C20:5**	-	-	-	-	-	-
**C22:0**	-	-	-	-	-	-
**C22:1**	-	-	-	-	-	-
**C22:6**	-	-	-	-	-	-
**C24:0**	-	-	-	-	-	-
**C24:1**	-	-	-	-	-	-
**Total**	369.3	± 14.59	619.04	± 33.8	100	100

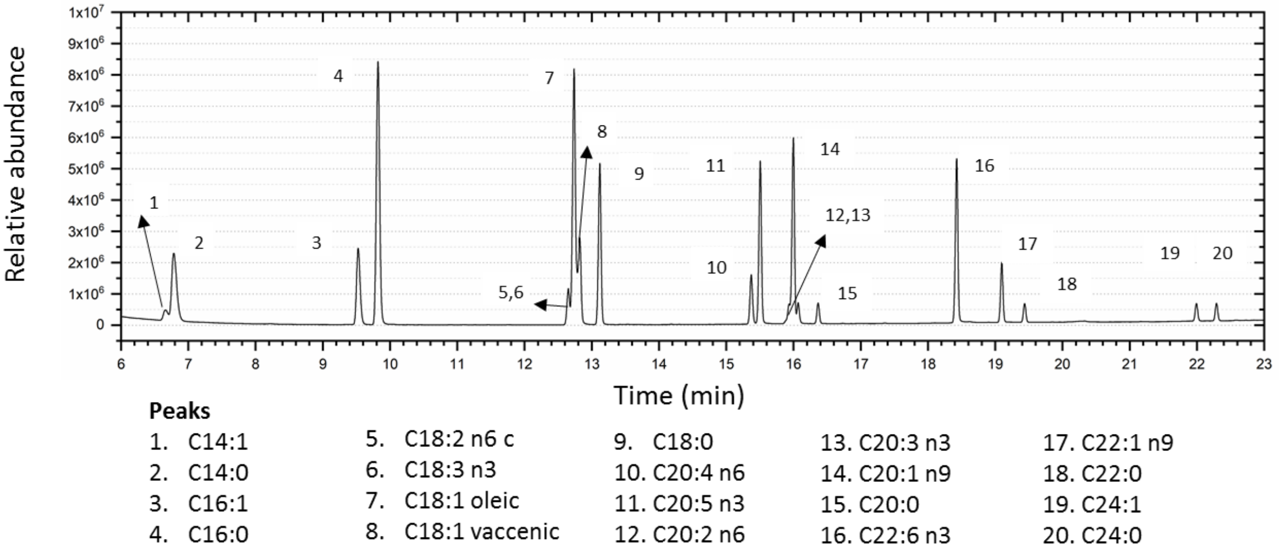

Figure S1. GC-FID chromatogram of the Marine Oil FAME Mix (RESTEK, USA) that was used as a standard reference in this study.

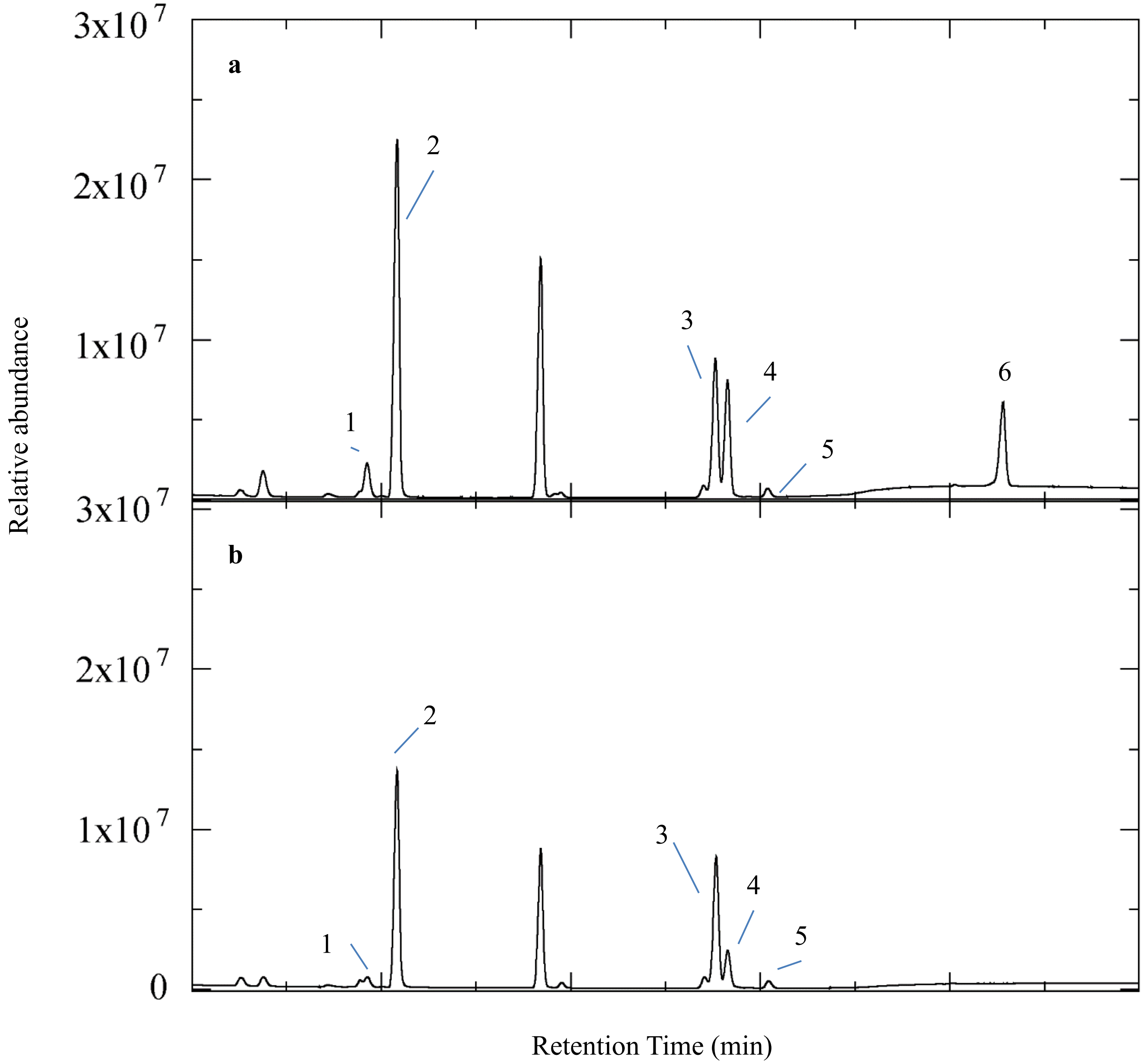

Figure S2. GC-FID chromatogram of methylated fatty acid samples acquired from fermentation cultures of BL21 (DE3) expressing pACYC-VLCFA-11GFP and pET28a-1-10GFP (a) and the control *Escherichia coli* BL21 (DE3) expressing pACYC-11-saGFP and pET28a-1-10saGFP (b). List of peaks: 1) C16:1; 2) C16:0; 3) C18:1 oleic; 4) C18:1 vaccenic; 5) C18:0; 6) Mixture of C20:0, C20:1and erucamide.

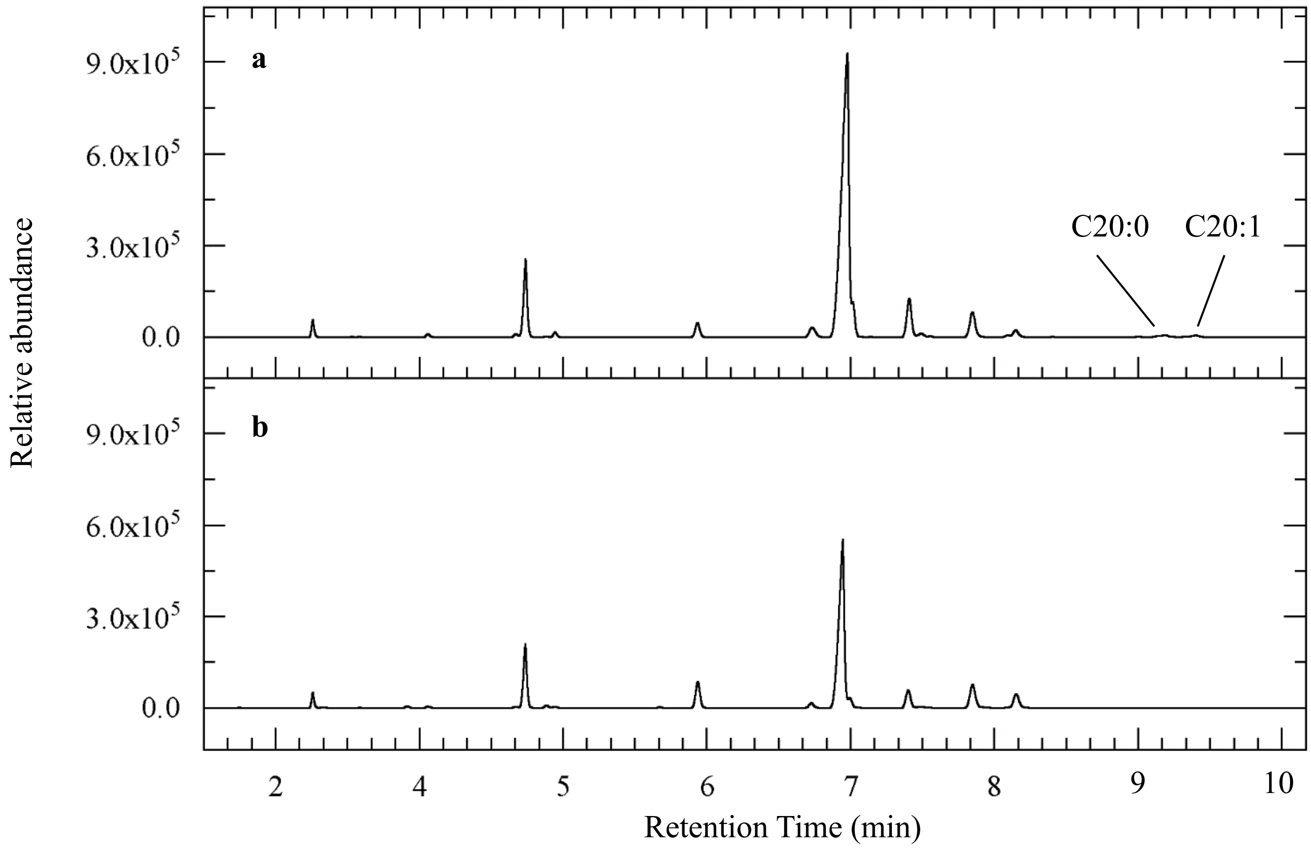

Figure S3. GC-FID chromatogram of methylated fatty acid samples acquired from fermentation cultures of BL21 (DE3) expressing pACYC-VLCFA-11GFP and pET28a-1-10GFP (a) and the control *Escherichia coli* BL21 (DE3) expressing pACYC-11-saGFP and pET28a-1-10saGFP (b). The list of peaks and retention times are listed in tables 6 and 7.

Shimadzu GC-2010 Plus gas chromatograph with flame ionization detector (FID) was used for this analysis. 1 µL sample was injected via an AOC-20i auto injector (Shimadzu) on to a Phenomenex ZB-WAX column (length 30 m, 0.32 mm ID, 0.25 µm df). The column was heated up with 5°C min-1 to 240°C maintained for 5 min. Hydrogen was used as carrier gas with a flow rate of 3 mL min-1 and constant flow compensation.

Table S6. List of fatty acid methyl ester peaks that are shown in figure 3 (a).

**Fatty acid methyl ester**	**R.Time**	**Area**	**Height**
**C16:0**	4,486	581536	254609
**C16:1**	4,732	37681	16244
**C18:0**	6,878	123096	33193
**C18:1 (vaccenic)**	7,216	196931	114231
**C20:0**	9,599	7039	1816
**C20:1**	9,822	43064	6393
**C19:0 Istd**	8,217	279403	82866

Table S7. List of fatty acid methyl ester peaks that are shown in figure 3 (b).

Fatty acid methyl ester	R.Time	Area	Height
**C16:0**	4,484	479004	210820
**C16:1**	4,732	15999	6738
**C18:0**	6,871	59105	18434
**C18:1 (vaccenic)**	7,192	82929	34395
**C20:0**	-	-	-
**C20:1**	-	-	-
**C19:0 Istd**	8,218	272617	78469

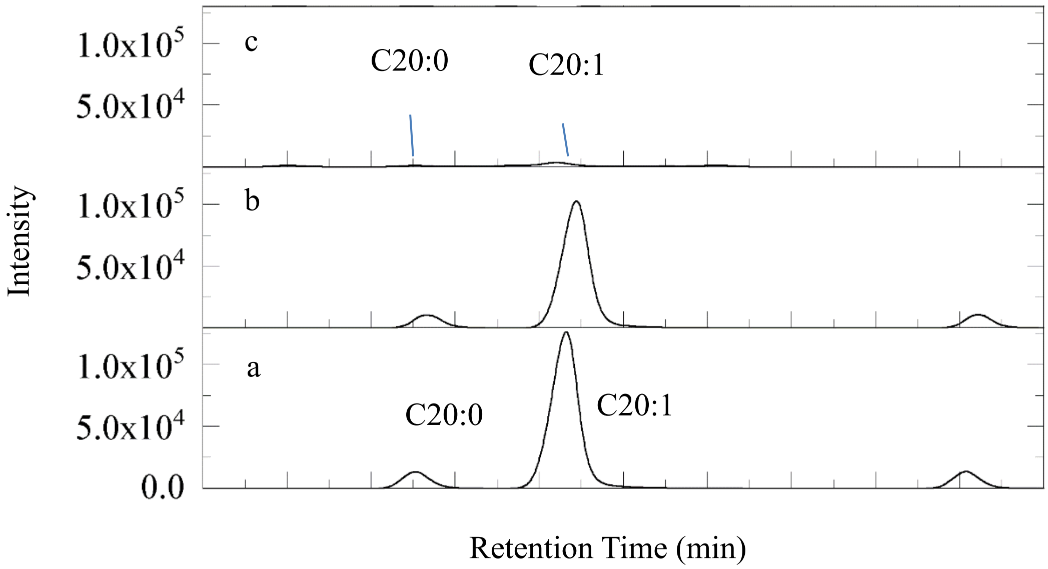

Figure S4. Enhanced GC-FID chromatogram of figure 3 representing the Arachidic (C20:0) and Eicosenoic (C20:1) FAME peaks of (a) and (b): Standards and (c): BL21 (DE3) expressing pACYC-VLCFA-11GFP and pET28a-1-10GFP.

References

Cabantous, S., Terwilliger, T.C., and Waldo, G.S. (2005). Protein tagging and detection with engineered self-assembling fragments of green fluorescent protein. *Nat Biotechnol* 23**,** 102-107.

Cabantous, S., and Waldo, G.S. (2006). In vivo and in vitro protein solubility assays using split GFP. *Nat Methods* 3**,** 845-854.

Espah Borujeni, A., Channarasappa, A.S., and Salis, H.M. (2014). Translation rate is controlled by coupled trade-offs between site accessibility, selective RNA unfolding and sliding at upstream standby sites. *Nucleic Acids Res* 42**,** 2646-2659.

Salis, H.M., Mirsky, E.A., and Voigt, C.A. (2009). Automated design of synthetic ribosome binding sites to control protein expression. *Nat Biotechnol* 27**,** 946-950.
